# Synthetic sulfonated derivatives of poly(allylamine hydrochloride) as inhibitors of human metapneumovirus

**DOI:** 10.1371/journal.pone.0214646

**Published:** 2019-03-28

**Authors:** Justyna Ciejka, Paweł Botwina, Maria Nowakowska, Krzysztof Szczubiałka, Krzysztof Pyrc

**Affiliations:** 1 Virogenetics Laboratory of Virology, Malopolska Centre of Biotechnology, Jagiellonian University, Krakow, Poland; 2 Faculty of Chemistry, Jagiellonian University, Krakow, Poland; 3 Department of Microbiology, Faculty of Biochemistry, Biophysics and Biotechnology, Jagiellonian University, Krakow, Poland; Arizona State University, UNITED STATES

## Abstract

Human metapneumovirus (hMPV) is a widely distributed pathogen responsible for acute upper and lower respiratory infections of varying severity. Previously, we reported that N-sulfonated derivatives of poly(allylamine hydrochloride) (NSPAHs) efficiently inhibit replication of the influenza virus *in vitro* and *ex vivo*. Here, we show a dose dependent inhibition of hMPV infection by NSPAHs in LLC-MK2 cells. The results showed strong antiviral properties of NSPAHs. While the activity of NSPAHs is comparable to those of carrageenans, they show better physicochemical properties and may be delivered at high concentrations. The functional assays showed that tested polymers block hMPV release from infected cells and, consequently, constrain virus spread. Moreover, further studies on viruses utilizing different egress mechanisms suggest that observed antiviral effect depend on selective inhibition of viruses budding from the cell surface.

## Introduction

Human metapneumovirus (hMPV) is associated with acute infections of the respiratory tract, with increased severity in children, infants, elderly, and immunocompromised patients [1–4]. hMPV was identified in 2001 in the Netherlands [5] and sorted to the *Pneumoviridae* family. Its prevalence in children and infants requiring hospitalization worldwide reaches 4–25% [6,7]. Moreover, serological studies showed that ~70% of children are seropositive at the age of five [8]. Symptoms associated with the infection are usually mild (e.g., cough, subfebrile temperature), but more severe disease also occurs (e.g., bronchitis, pneumonia) [9,10].

hMPV F protein utilizes heparan sulfate (HS), a negatively charged glycosaminoglycan (GAG) present on the cellular membrane, as an attachment factor [11]. Previous studies have shown that natural polysaccharides containing sulfonate groups efficiently inhibit infections caused by viruses employing HS during cell entry [12–19] and it was proposed that carrageenans inhibit the attachment of the hMPV to HS by interacting with the F protein [19]. It is, however, worth to mention that replication of some HS independent viruses is also hampered in the presence of these polymers [20–23]. Currently, in some countries, ι-carrageenan is the main component of nasal spray used to prevent respiratory viral infections [24,25].

In our previous report we showed that N-sulfonated derivatives of poly(allylamine) hydrochloride (NSPAHs) effectively inhibit influenza virus *in vitro* and *ex vivo* by inhibition of virion release from infected cells [26]. Surprisingly, we showed that new compounds share the mechanism of action with ι-carrageenan, and it is very different from previously proposed. Although the antiviral activity of NSPAHs is comparable to the one observed for carrageenans, physicochemical properties of NSPAHs seem to be superior [27].

The aim of this study was to examine the anti-hMPV activity of NSPAHs. For this, a series of *in vitro* experiments were conducted. Two NSPAH derivatives were selected for the study: NSPAH-15-94 (molecular mass (Mw) of 15 kDa, degree of substitution with sulfonate groups (DS) of 94%) and NSPAH-65-96 (Mw = 65 kDa, DS = 96%), as these were the most potent inhibitors of hMPV replication in the preliminary studies. As reference, two sulfonated polysaccharides with well-established antiviral properties (ι-carrageenan, and λ-carrageenan) were used.

## Materials and methods

### Polymers

NSPAH-15-94 and NSPAH-65-96 were synthesized, analyzed and purified by dialysis as described before [26]. The purification process was carried out against deionized water for 1 week (molecular mass cutoff of 14 kDa). Afterward, the water was removed by freeze drying to give the product as pale-yellow crystals. Iota carrageenan (ι-car) and lambda carrageenan (λ-car) were obtained from Sigma-Aldrich, Poland.

### Cell culture

Rhesus monkey kidney epithelial cells (LLC-MK2, ATCC CCL-7) were maintained in minimal essential medium (MEM) containing 1 part of Earle’s MEM and two parts of Hanks MEM (Thermo Scientific, Poland) supplemented with 3% heat-inactivated fetal bovine serum (FBS; PAA Laboratories, Austria), penicillin (100 U/ml) and streptomycin (100 μg/ml) (Sigma-Aldrich, Poland). Cells were propagated in T75 flasks (TPP, Switzerland) at 37°C in atmosphere containing 5% CO_2_.

### Virus preparation, titration and infection

hMPV virus stock (clinical isolate, clade B2) was kindly provided by Dr. Oliver Schildgen (Institute of Pathology, Witten/Herdecke University). hMPV A1 virus stock (strain: NL/1/00) was acquired from the European Virus Archive (011V-00930). Viruses were propagated as described before [28]. Human coronavirus NL63 (HCoV-NL63) stock was prepared as described before [17]. All assays were carried out using fully confluent LLC-MK2 (1.5 × 10^4^ cells per well) cultured for 48 h on 96-well plates (TPP, Switzerland). Cells were infected with virus at TCID_50_ (50% tissue culture infectious dose) of 400 per ml (MOI = 0.05) in 0% DMEM (Dulbecco’s Modified Eagle’s Medium (Thermo Scientific, Poland) deprived of FBS, supplemented with penicillin (100 U/ml), streptomycin (100 μg/ml), gentamicin (50 μg/ml), amphotericin (2.5 μg/ml) and trypsin (1 μg/ml) (Sigma-Aldrich, Poland)). Virus titers were assessed as described by Reed and Muench [28,29].

### XTT assay

Cell viability was evaluated using XTT Cell Viability Assay kit (Biological Industries, USA) according to the manufacturer’s instructions. Cells were incubated with tested polymers for 6 days at 37°C in atmosphere containing 5% CO_2_. After incubation, the medium was discarded, cells were rinsed with phosphate-buffered saline (PBS) and 100 μl of fresh medium was added to each well. Next, 50 μl of the activated 2,3-bis-(2-methoxy-4-nitro-5-sulphenyl)-(2H)-tetrazolium-5-carboxanilide (XTT) solution was added and samples were incubated for 2 h at 37°C. The absorbance (λ = 450 nm) was measured using Spectra MAX 250 spectrophotometer (Molecular Devices, USA).

### Quantitative real-time PCR

Virus yield was determined by reverse transcription (RT) quantitative real-time PCR (qPCR). To ensure that polymers do not affect the isolation and/or amplification the template, supernatants were diluted 1000 times and total RNA was isolated with Viral DNA/RNA kit (A&A Biotechnology, Poland) in accordance with the manufacturer’s instructions. Reverse transcription reaction was carried out with a high-capacity cDNA reverse transcription kit (Thermo Scientific, Poland) according to the manufacturer’s instructions. qPCR was performed with RT HS-PCR Mix Probe (A&A Biotechnology, Poland), as described before [28,30] (hMPV: primers (450 nM each) 5′ GCT GTC AGC TTC AGT CAA TTC AA 3′ and 5′ GTT ATC CCT GCA TTG TCT GAA AAC T 3′; probe (100 nM): 5′ 6-FAM-CGC ACA ACA TTT AGG AAT CTT CT-MGB 3′. HCoV-NL63: primers (450 nM each) 5′-AAA CCT CGT TGG AAG CGT GT-3′ and 5′-CTG TGG AAA ACC TTT GGC ATC-3′; probe (100 nM): 5′-FAM-ATG TTA TTC AGT GCT TTG GTC CTC GTG AT-TAMRA-3). The number of virus copies was determined using a series of 10-fold diluted DNA standards containing a known number of template copies. The reaction was carried out as follows: 2 min at 50°C, 10 min at 92°C, followed by 40 cycles of 15 s at 92°C and 1 min at 60°C. For each virus a standard curve was prepared separately. The lower limit of detection was 10^3^ viral RNA copies per milliliter.

### Replication inhibition assay

To study antiviral properties of the compounds, cells were infected with hMPV or inoculated with the mock sample in the presence or absence of polymer. After 2 h infection at 37°C, unabsorbed hMPV virions were removed by rinsing cells thrice with PBS. Subsequently, media supplemented with inhibitors were added and cultures were incubated for 6 days at 37°C. After incubation, culture supernatants were analyzed by titration and by RT-qPCR, as described above.

## Mechanism of action

To study the mechanism of action of tested polymers, a battery of functional assays were conducted as described before [26], with some modifications.

### Virus inactivation assay

The assay shows the influence of tested compounds on the viral particle; polymers at a final concentration of 1000 μg/ml were mixed with virus stock and incubated for 1 h at room temperature while mixing at 1000 rpm. hMPV stock incubated in the absence of the polymer was treated as a control. After incubation, samples were diluted 200 times to ensure that the polymer concentration is below the lower limit of the effective range. Samples were titrated as described by Reed and Muench [29].

### Cell protection assay

The assay shows the effect of polymers on the cell; a confluent culture of LLC-MK2 cells was inoculated with 100 μl of polymer solution (1000 μg/ml) per well. Samples were incubated at 37°C for 2 h, media were removed, and 100 μl virus or mock solution (MOI = 0.05) was added to each well. After 2 h incubation at 37°C media were discarded and cells were rinsed thrice with PBS to remove residual virus particles. Then, 100 μl of fresh medium was added to each well and plate was incubated for 6 days at 37°C.

### Virus attachment assay

The assay allows to examine the effect of the compound on the virus-receptor interaction; a confluent culture of LLC-MK2 cells was pre-cooled to 4°C and overlaid with 100 μl of media supplemented with tested polymer (1000 μg/ml) and virus (MOI = 0.05) or mock control. The samples were incubated at 4°C for 1 h to allow virus attachment but to block virus internalization [31]. After incubation, cells were rinsed thrice with ice-cold PBS to remove residual virus particles. Then, 100 μl of fresh medium was added and cells were incubated for 6 days at 37°C.

### Virus internalization assay

The assay allows to evaluate virus entry into the susceptible cell. Pre-cooled (4°C) confluent cells were infected with hMPV at MOI of 0.05 or treated with mock. The plate was incubated for 2 h at 4°C to allow virus attachment, but not internalization. Supernatants were discarded and cells were washed thrice with ice-cold PBS to remove unbound virus particles. Next, 100 μl of media supplemented with polymers (1000 μg/ml) was added and cells were incubated for 2 h at 37°C to allow virus internalization. Finally, the supernatants were discarded, cells were washed with an acidic buffer (pH 3.0, 0.1 M glycine, 0.1 M sodium chloride) to inactivate particles that did not enter the cell. Cultures were incubated for 6 days at 37°C in medium deprived of polymers.

### Virus replication, assembly and egress assay

The assay eEvaluates virus replication and production of infectious progeny. Confluent cells were incubated with 100 μl of the hMPV at MOI of 0.05. The infection was carried out for 2 h at 37°C. Subsequently, medium was discarded and cultures were rinsed thrice with PBS. Cells were overlaid with media supplemented with polymers or control samples and incubated for 6 days at 37°C. In this test, both supernatants and cell lysates were analyzed. Lysates were prepared by 3 fast cycles of freezing and thawing at -80°C in R9F buffer (A&A Biotechnology, Poland).

### Confocal microscopy

For confocal microscopy analysis, replication inhibition assay was performed on cells seeded on coverslips in 6-well plates (TPP, Switzerland). Subsequently, cells were washed with PBS, fixed with 4% paraformaldehyde, permeabilized with 0.1% Triton X-100, and incubated for 2 h with 5% bovine serum albumin (BSA) containing 0.1% Tween 20 (Sigma-Aldrich, Poland). Subsequently, cells were incubated for 2 h with 1 μg/ml mouse anti-hMPV F-protein antibody (Ingenasa, Spain). Then, cells were incubated for 1 h with 2.5 μg/ml Alexa Fluor 488-labeled goat anti-mouse antibody (Molecular Probes, USA). Nuclear DNA was stained with DAPI (0.1 μg/ml, Sigma-Aldrich, Poland). Fluorescent images were acquired with a Nikon Eclipse Ti-E confocal microscope under NIS-Elements—Microscope Imaging Software (Nikon Instruments Europe B.V., Amsterdam, The Netherlands).

### Statistical analysis

All experiments were conducted at least in triplicate. The results are expressed as means ± standard deviations (SD). The 50% inhibitory concentration (IC_50_) values were calculated using the Graph Pad Prism function. To determine the significance of the results obtained, Student *t*-test was used and P values < 0.05 were considered significant. Virus yields and virus titers are presented as normalized data, i.e., log reduction values (LRVs) [26] for easier comparison of the inhibitory effect between assays. LRV was calculated according to the following formula: LRV = log (c_i_/c_0_) where c_i_ is the number of viral RNA copies per milliliter in the sample treated with inhibitor and c_0_ is the number of viral RNA copies per milliliter in control sample (untreated cells). Raw research data (number of copies per milliliter or TCID_50_ values) are presented in supporting information file.

## Results and discussion

### Cytotoxicity of polymers on LLC-MK2 cells

**Fig 1:**
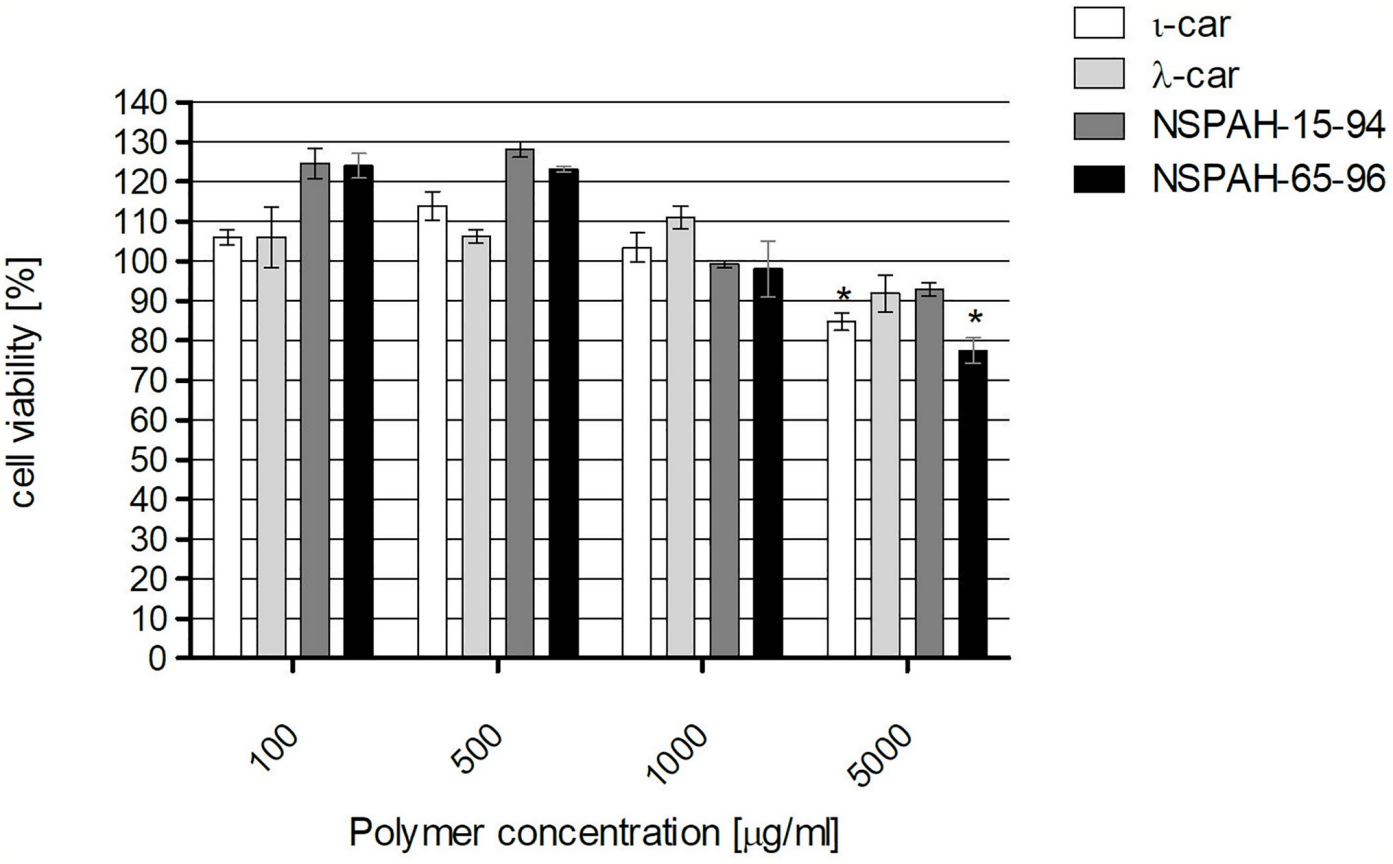
Sulfonated polymers are not toxic for cells at effective concentration range. The cytotoxicity of the tested polymers (ι-car: ι-carrageenan, λ-car: λ-carrageenan) determined by XTT assay. Values that are significantly different (P < 0.05) from the control are indicated by an asterisk. All experiments were performed in triplicate. Average values with standard deviations (error bars) are presented.

The cells were incubated for 6 days in the presence or absence of evaluated compound, and the cell viability was assessed using an XTT assay. While some cytotoxicity was observed for ι-car and NSPAH-65-96 at 5000 μg/ml (~80% of the control), at lower concentrations the viability of cells was not affected (Fig 1). Obtained results are consistent with previously published data [26].

### NSPAHs and carrageenans inhibit hMPV replication *in vitro*

**Fig 2:**
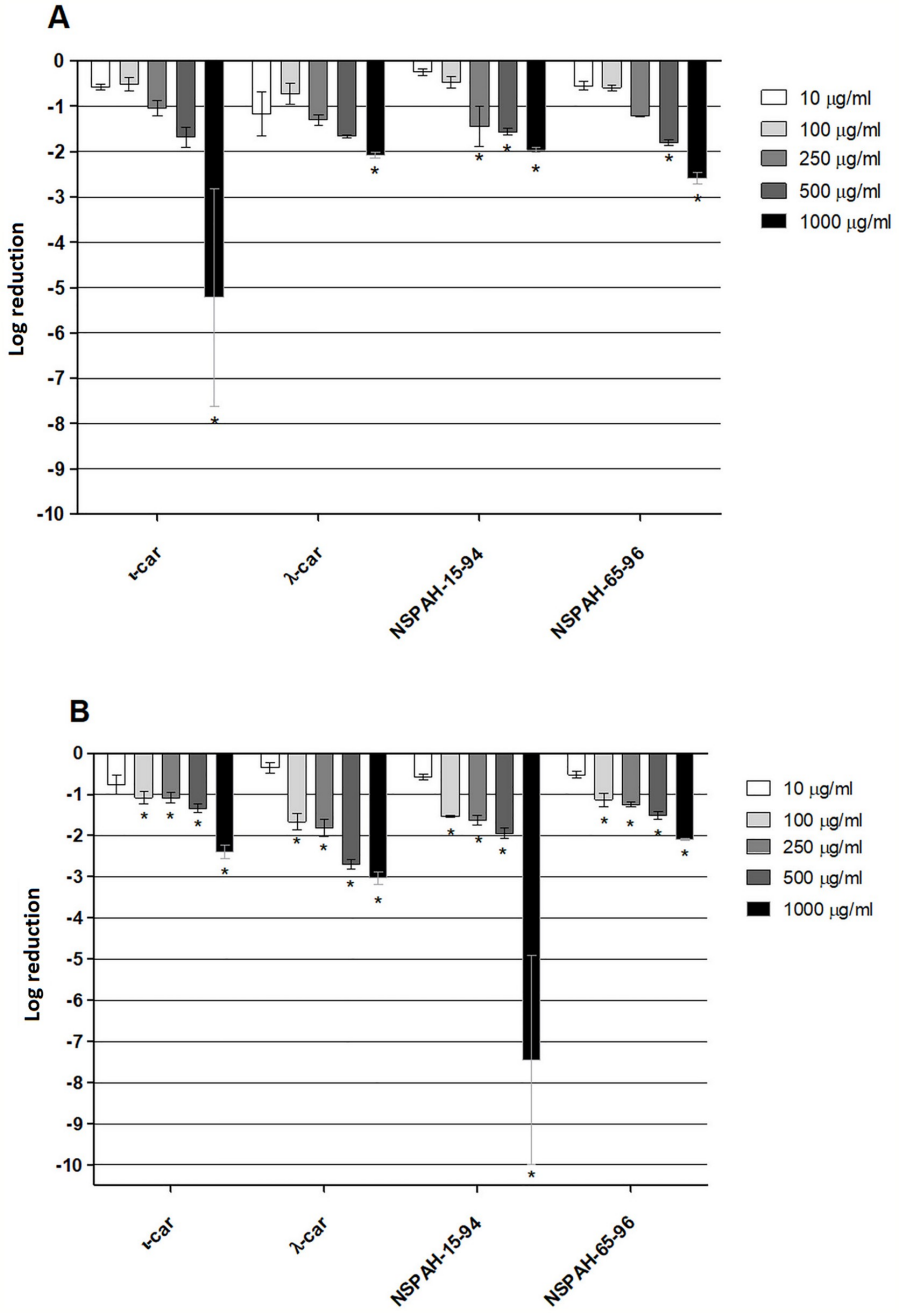
Sulfonated polymers hamper hMPV B2 infection. Inhibition of the infection was evaluated using RT-qPCR (**A**) and virus titration (**B**). Polymers were present during the whole infection process. Data are shown as log reduction values (LRVs) normalized to the control samples. All experiments were performed in triplicate. The results are presented as average values with standard deviations (error bars). An asterisk (P < 0.05) indicates values that are significantly different from the control. ι-car: ι-carrageenan, λ-car: λ-carrageenan.

**Fig 3:**
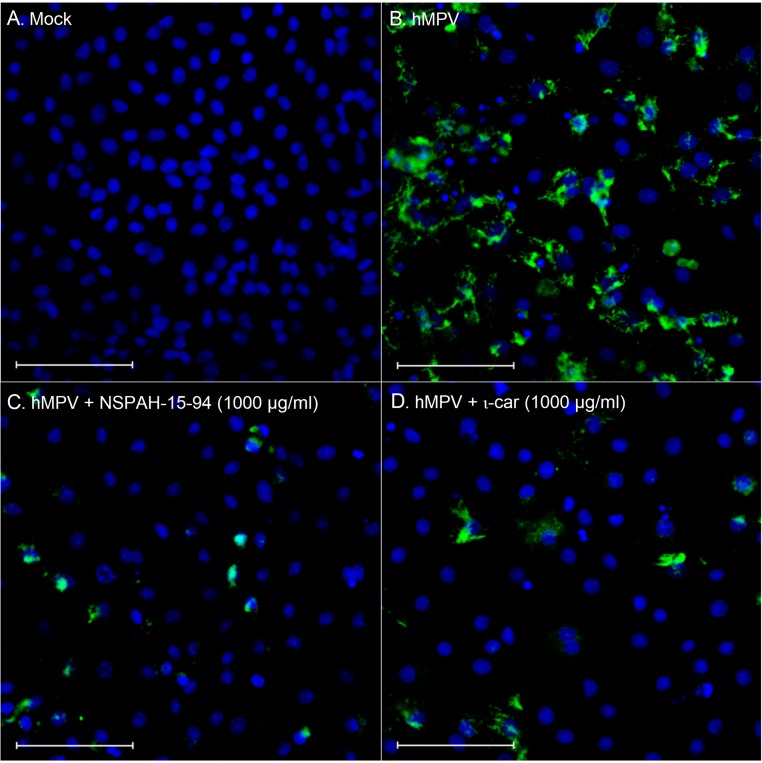
Sulfonated polymers limit the number of infected cells. NSPAHs and carrageenans inhibit viral infection by hampering virus release from infected cells. Fluorescence images of LLC-MK2 cells (nuclei in blue) infected with hMPV B2 (green) were collected 5 days p.i. (scale bar = 100 μm).

In the initial experiment, polymers were tested for their ability to hamper hMPV replication in LLC-MK2 cells. Cells were overlaid with media supplemented with polymers or control samples and hMPV or mock samples. Polymers were present during whole experiment. All compounds suppressed virus replication (RT-qPCR) by at least 2 log units at 1000 μg/ml, with IC_50_ values of 231 μg/ml, 236 μg/ml, 117 μg/ml, 166 μg/ml for ι-car, λ-car, NSPAH-15-94, and NSPAH-65-96, respectively (Fig 2A; S1 Fig). To test if infectivity of progeny virus is also diminished, cell culture supernatants were titrated. As visible in Fig 2B, NSPAH-15-94 exhibits the best antiviral properties. Confocal imaging confirmed this observation, as in the presence of the polymers the number of infected cells was lower compared to the control (Fig 3).

**Fig 4:**
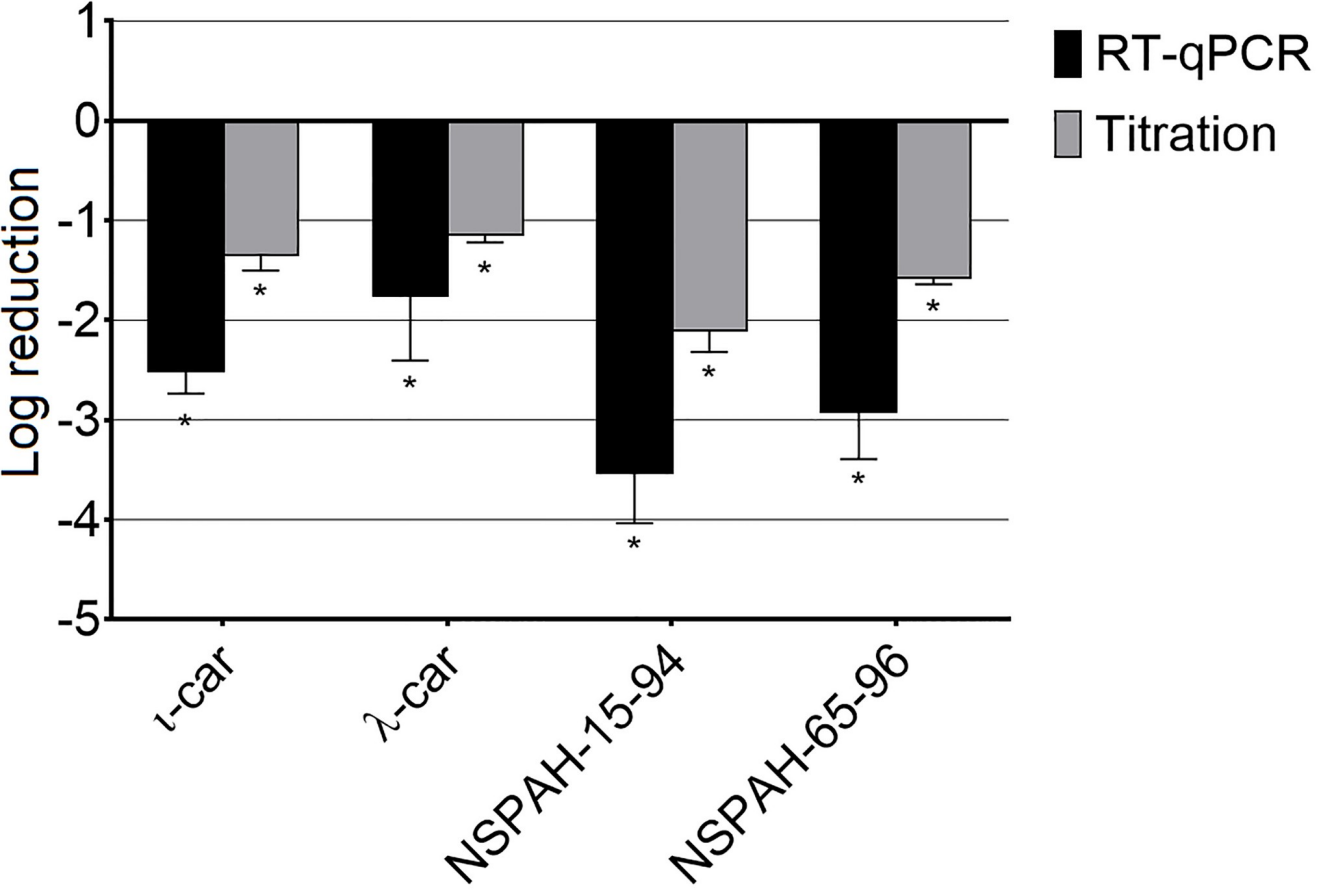
Sulfonated polymers hamper hMPV A1 infection. Inhibition of the infection was evaluated using RT-qPCR (black bars) and virus titration (grey bars). Polymers (1000 μg/ml) were present during the entire hMPV replication cycle. Data are shown as log reduction values (LRVs) normalized to the control samples. All experiments were performed in triplicate. The results are presented as average values with standard deviations (error bars). An asterisk (P < 0.05) indicates values that are significantly different from the control. ι-car: ι-carrageenan, λ-car: λ-carrageenan.

To ensure broad specificity of NSPAH towards different hMPV strains, the study was performed also using different hMPV strain (clade A1). Similar antiviral effect was observed (Fig 4; S8 Fig).

### NSPAHs and carrageenans hamper release of progeny virions from infected cells

**Fig 5:**
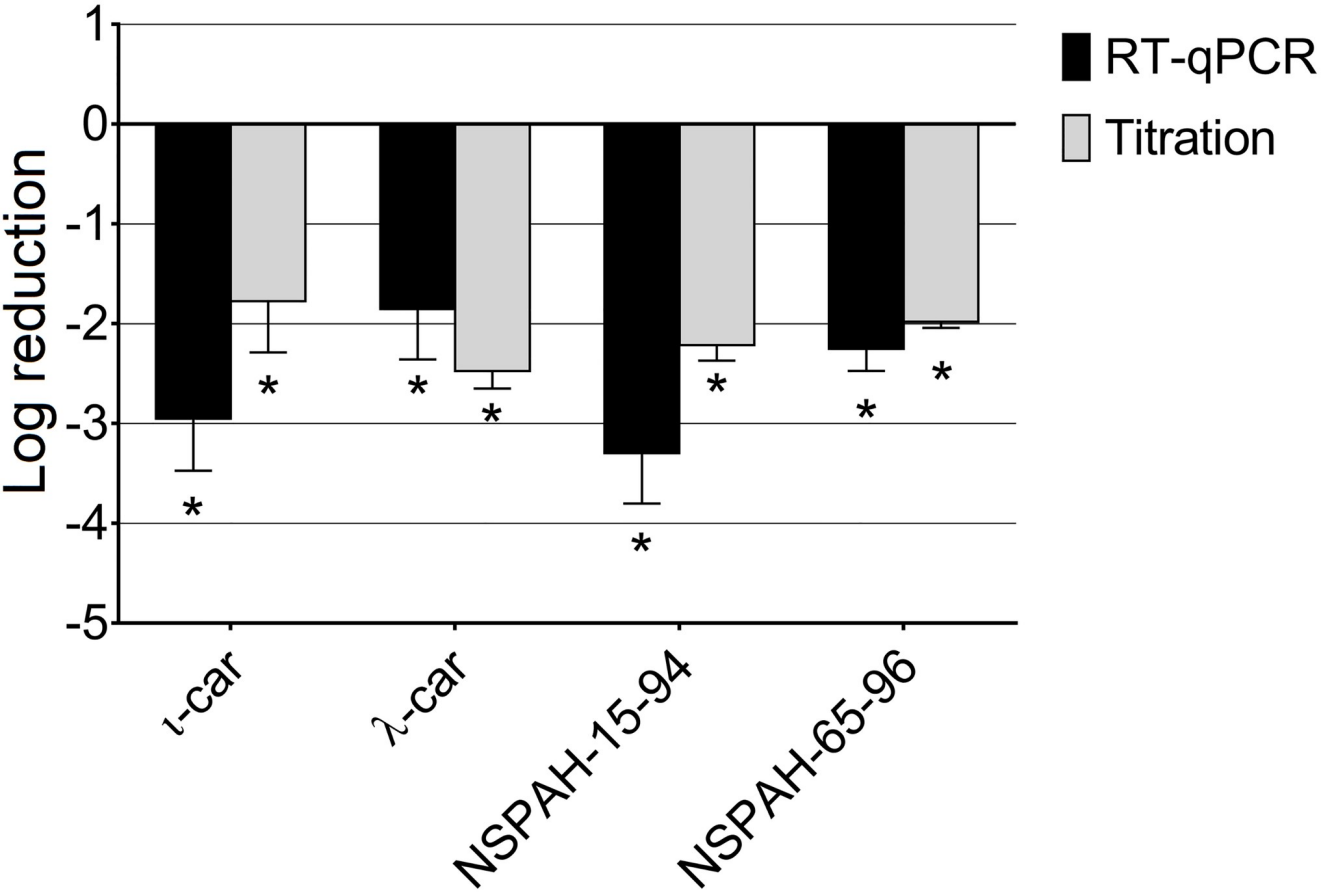
Inhibition of hMPV B2 replication by sulfonated polymers during late steps of the virus replication cycle. Inhibition of the infection was evaluated using RT-qPCR (black bars) and virus titration (grey bars). Polymers (1000 μg/ml) were added after infection of LLC-MK2 cells with hMPV for 2 h and supernatants were collected 6 days p.i. Data are shown as log reduction values (LRVs) normalized to the control samples. All experiments were performed in triplicate. The results are presented as average values with standard deviations (error bars). An asterisk (P < 0.05) indicates values that are significantly different from the control. ι-car: ι-carrageenan, λ-car: λ-carrageenan.

**Fig 6:**
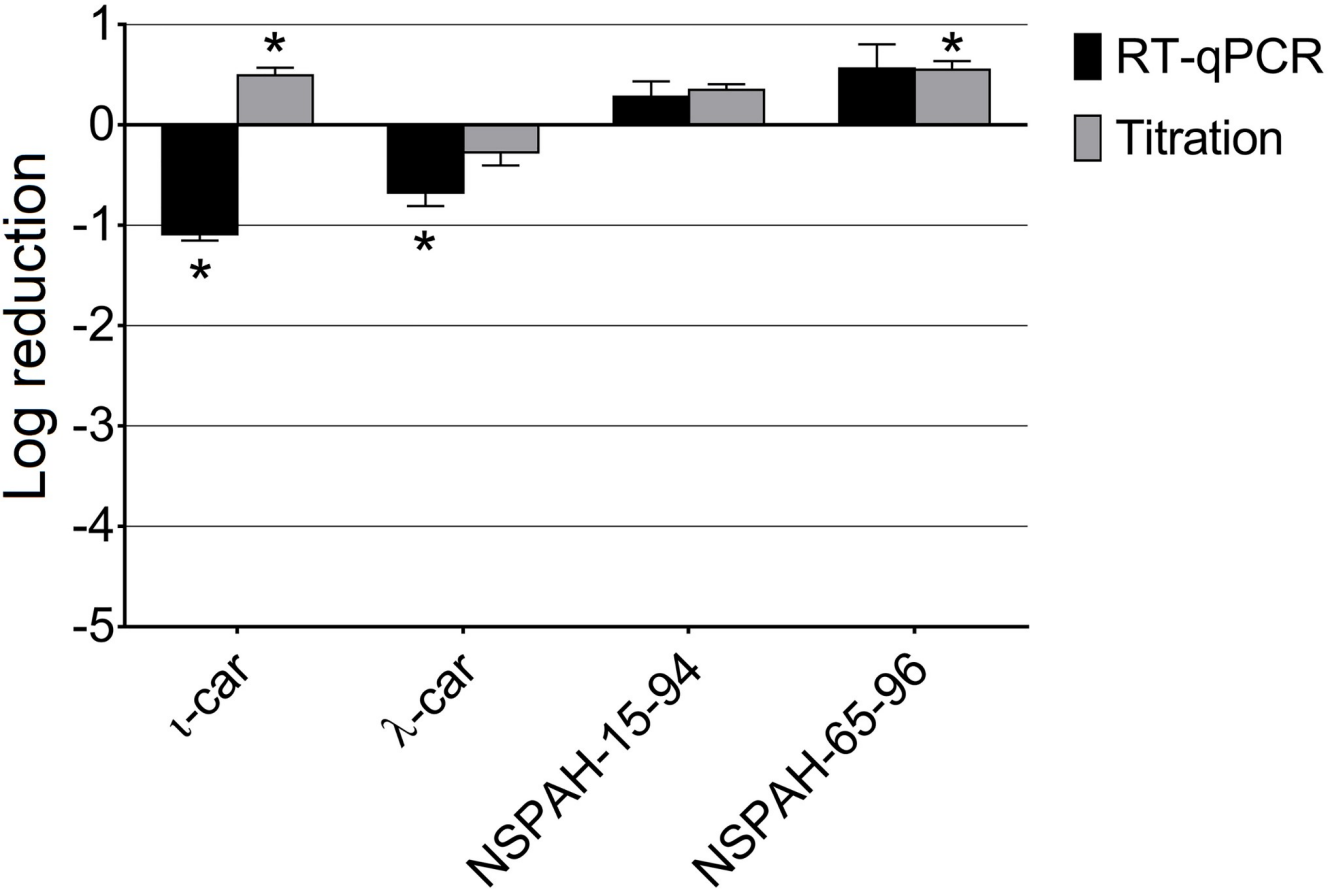
Sulfonated polymers do not inhibit intracellular hMPV B2 replication. Inhibition of intracellular hMPV replication was tested using RT-qPCR (black bars) or titration (grey bars). Polymers (1000 μg/ml) were added 2 h after infection of LLC-MK2 with hMPV and lysates were prepared 6 days p.i. Data are shown as log reduction values (LRVs) normalized to the control samples. All experiments were performed in triplicate. The results are presented as average values with standard deviations (error bars). An asterisk (P < 0.05) indicates values that are significantly different from the control. ι-car: ι-carrageenan, λ-car: λ-carrageenan.

In the next step, we aimed to elucidate the mechanism of action for described polymers. At 1000 μg/ml no inhibition of early steps of virus infection was noted for carrageenans or NSPAHs (first four functional assays, S4–S7 Figs), but we observed strong inhibition of progeny virus production in assay 5, wherein the inhibitors were added 2 h after the inoculation. All tested compounds inhibited hMPV progeny virus production, as illustrated by decreased virus yields and titers (p < 0.05). RT-qPCR analysis indicated that the inhibition is the most profound (ca. 3 log units) for ι-car and NSPAH-15-94 (Fig 5, black bars; S2 Fig). Titration of supernatants revealed that all compounds inhibited production of hMPV infectious virions by ca. 2 log units (Fig 5, grey bars; S2 Fig). Obtained results suggest that tested polymers hamper late stages of virus replication cycle, after the virus enters the cell. To further investigate the mechanism, supernatants from infected cultures were discarded and cells were lysed by three freeze and thaw cycles (titration) or with lysis buffer (RNA isolation). Surprisingly, RT-qPCR analysis revealed only minor inhibition (p < 0.05) of viral RNA replication by carrageenans and no inhibitory activity for NSPAHs (Fig 6, black bars; S3 Fig). This is consistent with our previous results on the influenza virus [26]. In the case of titration, we observed slight but significant increase in the infectivity of the samples in case of ι-car and NSPAH-65-96. NSPAH-15-94 also increase the infectivity of cell lysates, however, this increase was statistically non-significant (Fig 6, grey bars; S2 Fig). This indicates that hMPV virions correctly progress to the budding stage, but in the presence of the polymers they remain cell-associated. Altogether, obtained data indicate that NSPAHs do not affect virus replication *per se*, but lock them on cells and limit their spread to the neighboring cells.

While some previous reports suggest inhibition of early stages of viral replication by carrageenans [19], we were not able to replicate these results. It is, however, possible that the mechanism of action may very depending on the origin of these natural polymers [15,20,21,26].

**Fig 7:**
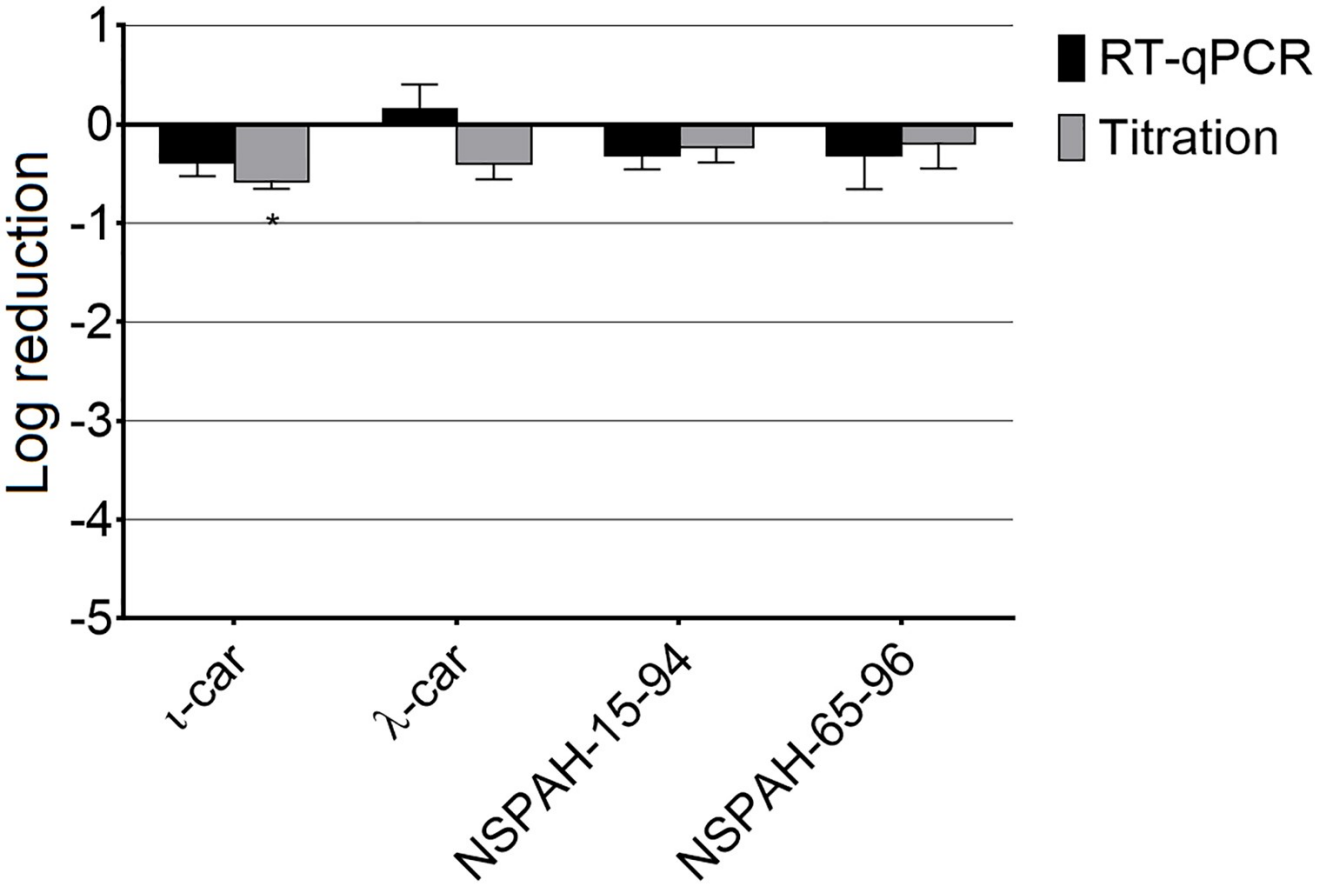
Sulfonated polymers do not inhibit HCoV-NL63 infection. Inhibition of the infection was evaluated using RT-qPCR (black bars) and virus titration (grey bars). Polymers (1000 μg/ml) were present throughout the infection. Data are shown as log reduction values (LRVs) normalized to the control samples. All experiments were performed in triplicate. The results are presented as average values with standard deviations (error bars). An asterisk (P < 0.05) indicates values that are significantly different from the control. ι-car: ι-carrageenan, λ-car: λ-carrageenan.

To study whether the observed antiviral effect is virus-specific, the same cell line was infected with an unrelated virus, i.e., human coronavirus NL63 (HCoV-NL63). This virus efficiently replicates in LLC-MK2 cells but is released from the cell in a different mechanism. In contrast to hMPV and influenza A viruses, which bud from the cell surface, HCoV-NL63 buds from the Golgi apparatus and mature infectious viral particles are transported *via* exocytosis to the cell surface [32]. None of the tested polymers inhibited HCoV-NL63 (Fig 7; S9 Fig). This observation suggests that the polymers block virus release from the cell surface, e.g., by modification of membrane plasticity or anchoring the progeny virions to the cell. One may also consider that the difference in NSPAHs activity between hMPV and HCoV-NL63 results from different requirements for the HSPGs presence, but it would not explain strong inhibitory effect of NSPAHs against the influenza virus. Moreover, HCoV-NL63 was also shown to employ HSPGs during the entry to the cell [33].

## Conclusions

To sum up, we demonstrated a dose-dependent inhibition of hMPV replication by sulfonated poly(allylamine)-based polymers. Among tested polymers, ι-carrageenan and NSPAH-15-94 show the strongest antiviral properties against hMPV. Studies on the mechanism of anti-hMPV properties of tested polymers imply that the decrease of samples’ infectivity is due to blocking virus release from the cellular membrane.

Developed synthetic polymers are not toxic and offer similar antiviral properties as carrageenans but show better physicochemical properties. In our previous work we reported that NSPAHs hamper influenza virus replication *in vitro* and *in vivo* and we believe that they may be considered as broad-spectrum drug candidates competitive to marketed compounds.

## Supporting information

S1 Fig Polymers hamper hMPV B2 infection. (PDF)

S2 Fig Inhibition of human metapneumovirus B2 virus (hMPV) replication in LLC-MK2 cells on late steps of virus replication. (PDF)

S3 Fig Polymers inhibit releasing step of the hMPV replication cycle. (PDF)

S4 Fig Virus inactivation assay (1). (PDF)

S5 Fig Virus adsorption assay (2). (PDF)

S6 Fig Virus attachment assay (3). (PDF)

S7 Fig Virus internalization assay (4). (PDF)

S8 Fig Sulfonated polymers hamper hMPV A1 infection. (PDF)

S9 Fig Sulfonated polymers do not inhibit HCoV-NL63 infection. (PDF)
