# Supplementary material for: Synthetic sulfonated derivatives of poly(allylamine hydrochloride) as inhibitors of human metapneumovirus
Source: PLoS One. 2019 Mar 28;14(3):e0214646. doi: 10.1371/journal.pone.0214646 (PMC6438514; doi:10.1371/journal.pone.0214646)
Supplement: S2 Fig — (PDF) [file pone.0214646.s002.pdf]

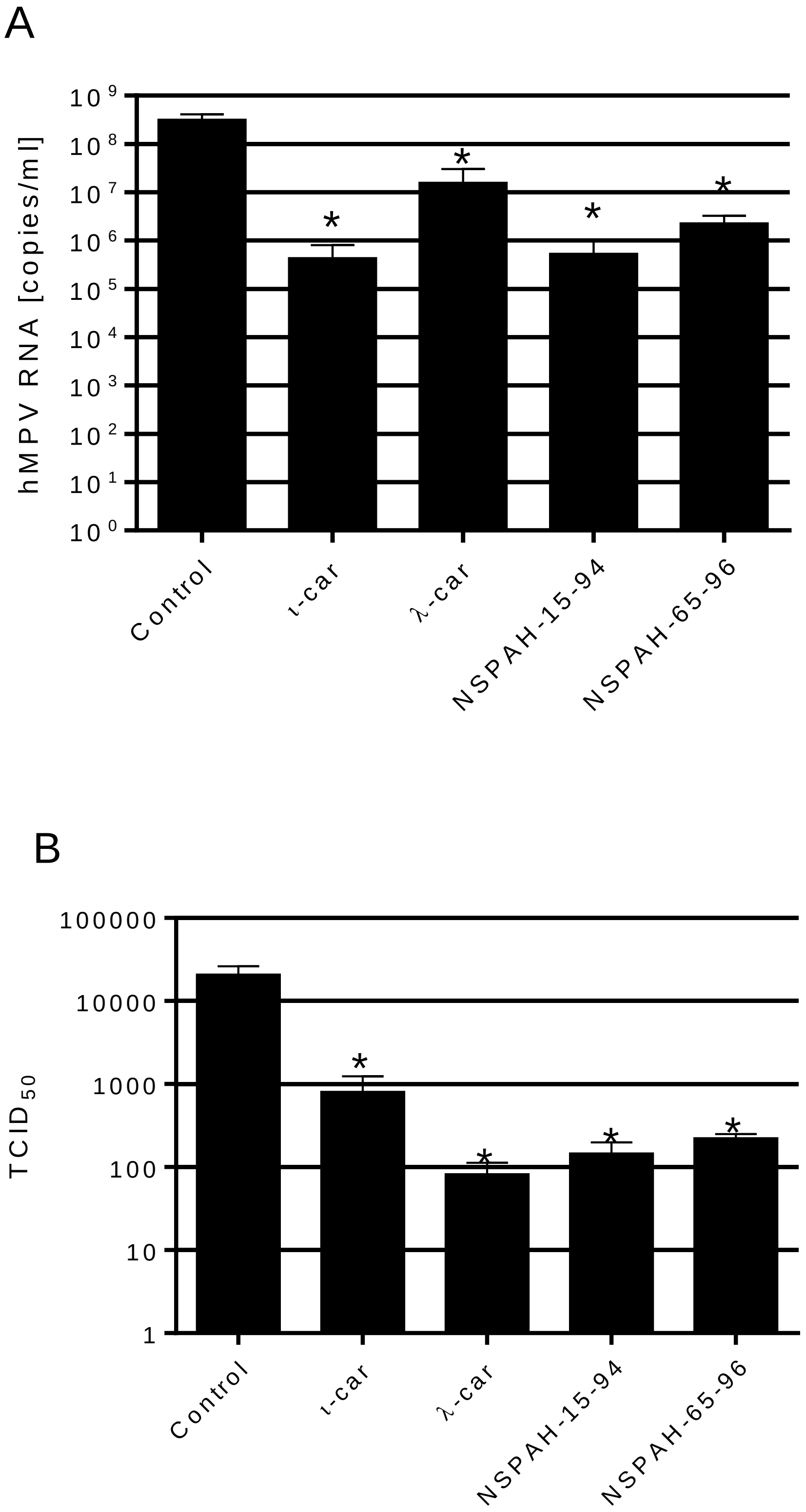

2S Fig. **Inhibition of human metapneumovirus B2 virus (hMPV) replication in LLC-MK2 cells on late steps of virus replication.** Polymers were added after infection of LLC-Mk2 cells with hMPV and supernatants were analyzed 6 days p.i. Inhibition is expressed as (A) decrease of viral RNA copies measured by quantitative real-time PCR and (B) decrease of virus titers expressed by Reed&Muench titration[1]. Values that are significantly different ($P < 0.05$) from the control are indicated by an asterisk. All experiments were performed in triplicate. Average values with standard deviations (error bars) are presented.
