# Supplementary material for: Synthetic sulfonated derivatives of poly(allylamine hydrochloride) as inhibitors of human metapneumovirus
Source: PLoS One. 2019 Mar 28;14(3):e0214646. doi: 10.1371/journal.pone.0214646 (PMC6438514; doi:10.1371/journal.pone.0214646)
Supplement: S3 Fig — (PDF) [file pone.0214646.s003.pdf]

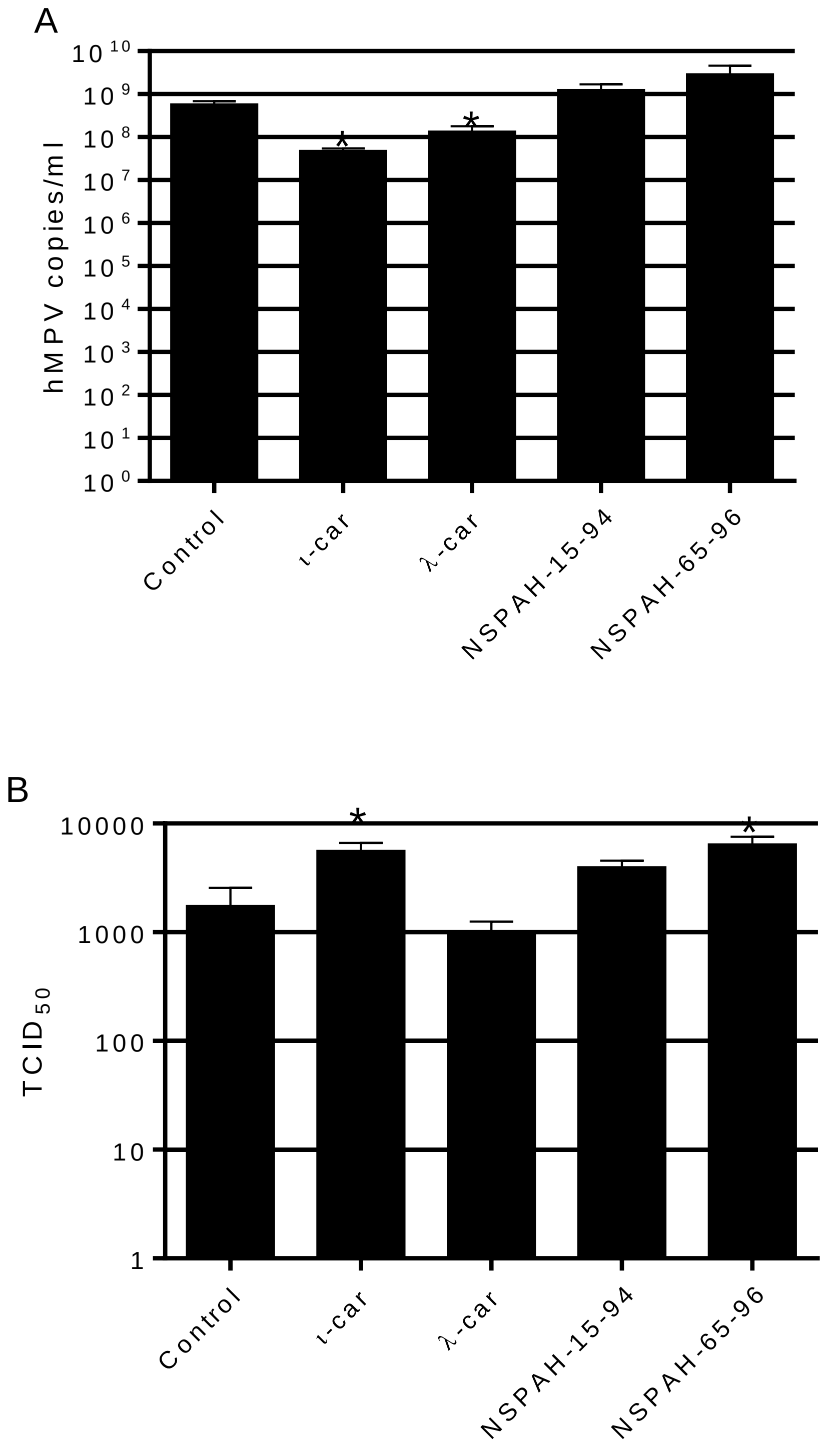

3S Fig. Polymers inhibit releasing step of the hMPV replication cycle. Charts show analysis of cellular lysates. Polymers; ι-carrageenan (ι -car), λ-carrageenan (λ -car), NSPAH-15-94 and NSPAH-65-96. were added after infection of LLC-MK2 with hMPV. Before lysis, supernatants were removed and new medium was applied. Lysates were made by three cycles of fast freezing and thawing. Results are expressed as (A) differences of viral RNA copies measured by quantitative real-time PCR and (B) virus titers expressed by Reed& Muench titration[1]. Values that are significantly different ($P < 0.05$) from the control are indicated by an asterisk. All experiments were performed in triplicate. Average values with standard deviations (error bars) are presented.
