# Supplementary material for: Synthetic sulfonated derivatives of poly(allylamine hydrochloride) as inhibitors of human metapneumovirus
Source: PLoS One. 2019 Mar 28;14(3):e0214646. doi: 10.1371/journal.pone.0214646 (PMC6438514; doi:10.1371/journal.pone.0214646)
Supplement: S4 Fig — (PDF) [file pone.0214646.s004.pdf]

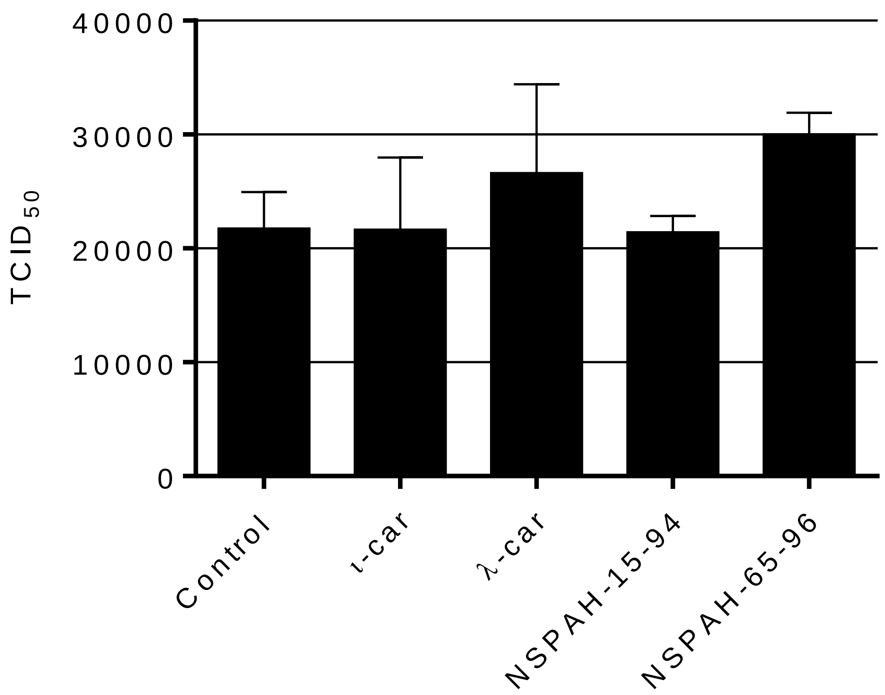

4S Fig. Virus inactivation assay (1) Inhibition of human metapneumovirus virus B2 (hMPV) replication in LLC-MK2. hMPV was pre-incubated with ι-carrageenan (ι -car), λ-carrageenan (λ -car), NSPAH-15-94 and NSPAH-65-96 before infection. Virus titers are expressed by Reed&Muench titration. Values that are significantly different ($P < 0.05$) from the control are indicated by an asterisk. All experiments were performed in triplicate. Average values with standard deviations (error bars) are presented.
