# Supplementary material for: Synthetic sulfonated derivatives of poly(allylamine hydrochloride) as inhibitors of human metapneumovirus
Source: PLoS One. 2019 Mar 28;14(3):e0214646. doi: 10.1371/journal.pone.0214646 (PMC6438514; doi:10.1371/journal.pone.0214646)
Supplement: S5 Fig — (PDF) [file pone.0214646.s005.pdf]

A

B

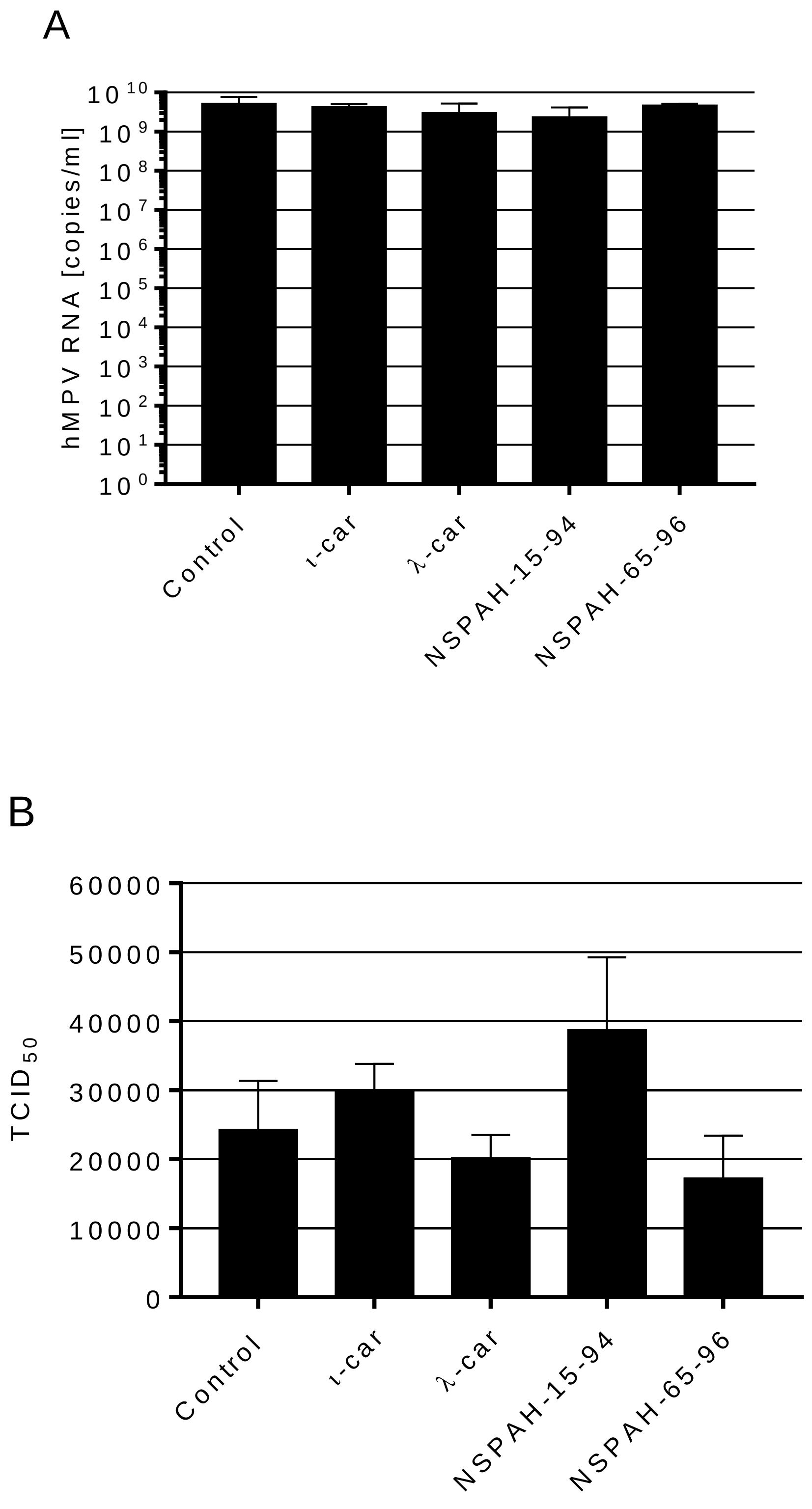

5S Fig. Virus adsorption assay (2). LLC-MK2 cells were incubated with ι-carrageenan (ι -car), λ-carrageenan (λ -car), NSPAH-15-94 and NSPAH-65-96 before infection with hMPV. Supernatants were analyzed 6 days p.i. Virus replication is expressed as (A) decrease of viral RNA copies measured by quantitative real-time PCR and (B) decrease of virus titers expressed by Reed&Muench titration. Values that are significantly different ($P < 0.05$) from the control are indicated by an asterisk. All experiments were performed in triplicate. Average values with standard deviations (error bars) are presented.
