# Supplementary material for: Synthetic sulfonated derivatives of poly(allylamine hydrochloride) as inhibitors of human metapneumovirus
Source: PLoS One. 2019 Mar 28;14(3):e0214646. doi: 10.1371/journal.pone.0214646 (PMC6438514; doi:10.1371/journal.pone.0214646)
Supplement: S7 Fig — (PDF) [file pone.0214646.s007.pdf]

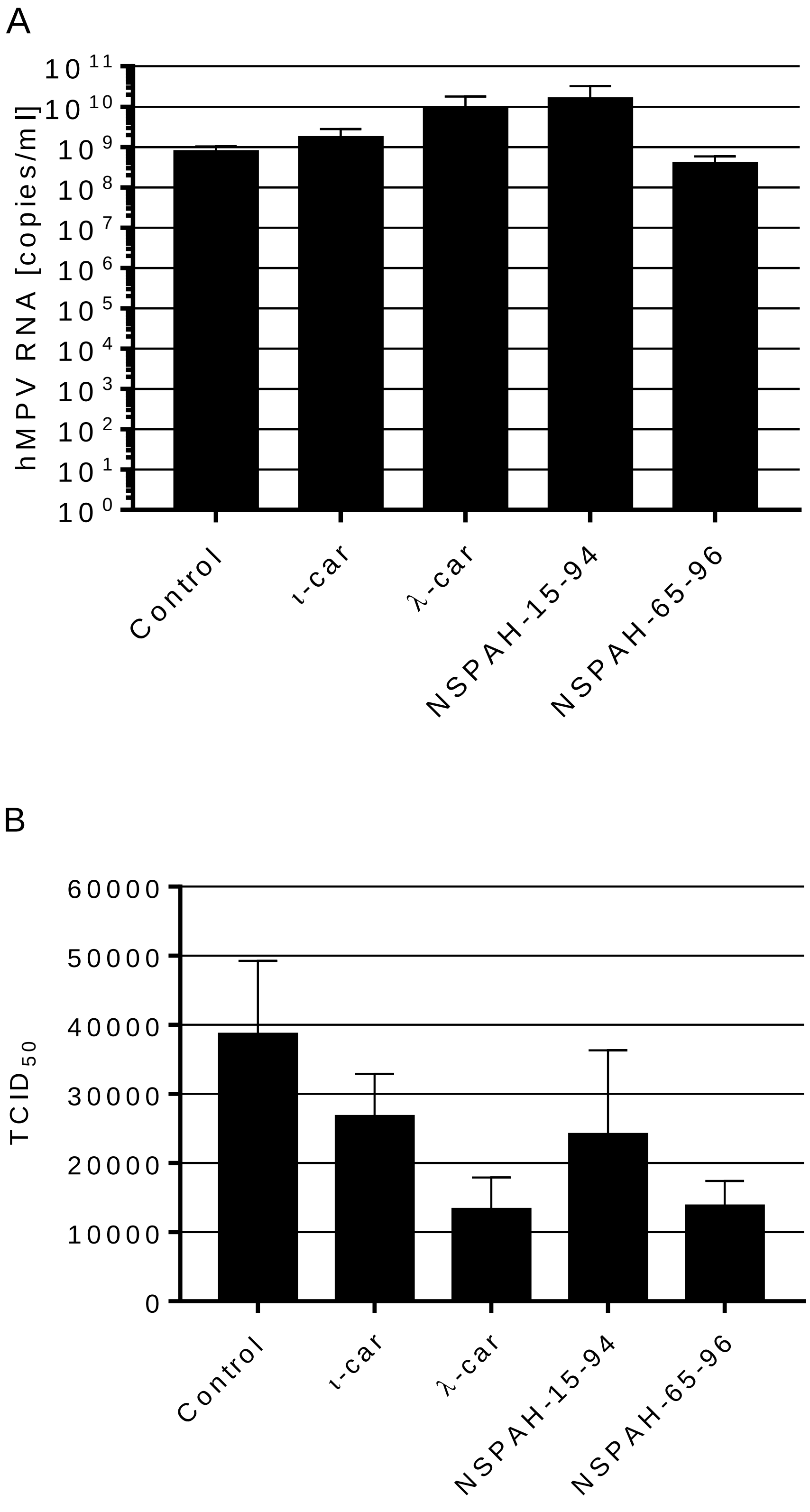

7S Fig. Virus internalization assay (4). LLC-MK2 cells were infected with hMPV at 4°C. Then, ι-carrageenan (ι -car), λ-carrageenan (λ -car), NSPAH-15-94 or NSPAH-65-96 were added and incubated 2 h in 37°C and fresh medium was applied. Supernatants were analyzed 6 days p.i. Virus replication is expressed as (A) viral RNA copies measured by quantitative real-time PCR and (B) virus titers expressed by Reed&Muench titration. Values that are significantly different ($P < 0.05$) from the control are indicated by an asterisk. All experiments were performed in triplicate. Average values with standard deviations (error bars) are presented.
