# Supplementary material for: Synthetic sulfonated derivatives of poly(allylamine hydrochloride) as inhibitors of human metapneumovirus
Source: PLoS One. 2019 Mar 28;14(3):e0214646. doi: 10.1371/journal.pone.0214646 (PMC6438514; doi:10.1371/journal.pone.0214646)
Supplement: S8 Fig — (PDF) [file pone.0214646.s008.pdf]

A

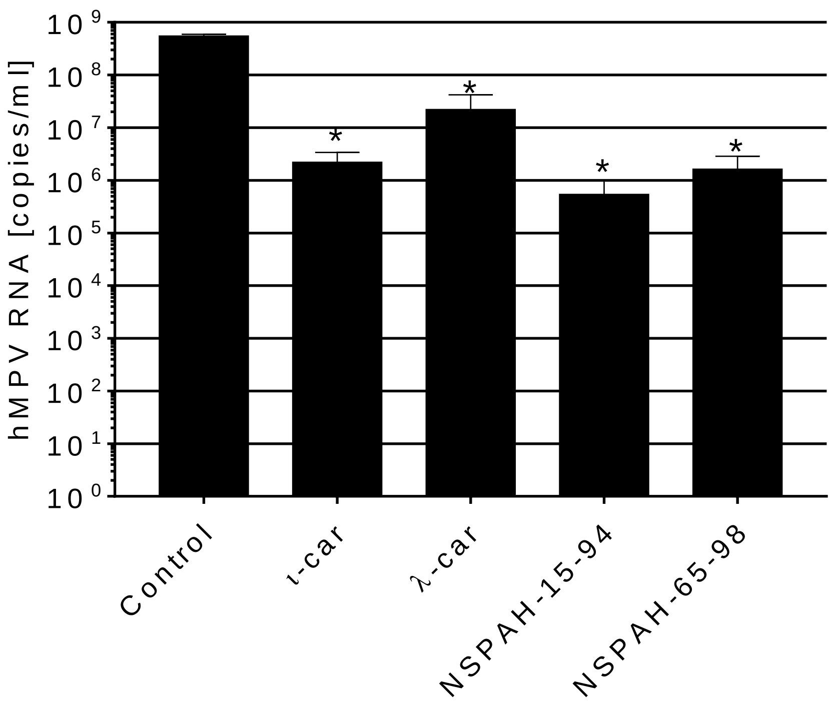

B

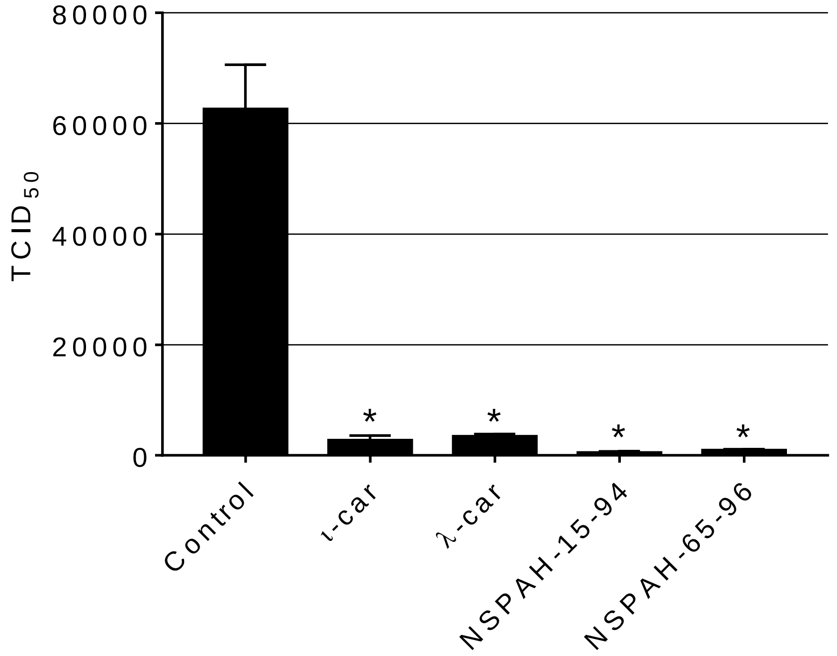

8S Fig. Sulfonated polymers hamper hMPV A1 infection. Virus inhibition was tested using RT-qPCR (**A**) and virus titration (**B**). Polymers (1000 μg/ml) were present during the whole infection. All experiments were performed in triplicate. The results are presented as average values with standard deviations (error bars). An asterisk ($P < 0.05$) indicates values that are significantly different from the control. ι-car: ι-carrageenan, λ-car: λ-carrageenan.
